# CLINICAL FACTORS ASSOCIATED WITH MOTOR IMAGERY ABILITY IN INDIVIDUALS WITH SUBACUTE STROKE

**DOI:** 10.2340/jrm.v58.46042

**Published:** 2026-08-17

**Authors:** Yuki FUKUMOTO, Tsubasa KAWASAKI, Takanao SHIRAI, Tomomi HASE, Hinata CHIYO, Hiroyuki HAMADA, Katsuya SAKAI, Junpei TANABE, Kota SAWA, Toshiaki SUZUKI

**Affiliations:** 1 Graduate School of Health Sciences, Graduate School of Kansai University of Health Sciences, Osaka; 2 Department of Physical Therapy, School of Health Sciences, Tokyo International University, Saitama; 3 Rehabilitation Center, Kiba Hospital, Medical Corporation, Juzankai, Osaka; 4 Department of Human and Engineered Environmental Studies, Graduate School of Frontier Sciences, The University of Tokyo, Tokyo; 5 Graduate School of Human Health Sciences, Tokyo Metropolitan University, Tokyo; 6 Department of Physical Therapy, Hiroshima Cosmopolitan University, Hiroshima; 7 Department of Physical Therapy, Faculty of Health Sciences, SBC Tokyo Medical University, Chiba, Japan

**Keywords:** memory, motor imagery, proprioception, stroke, upper extremity

## Abstract

**Objective:**

To examine the associations of motor recovery stage, proprioceptive function, and working memory with motor imagery ability in patients with subacute stroke.

**Design:**

Multicentre cross-sectional study.

**Subjects/Patients:**

Forty patients with subacute stroke who had complete data.

**Methods:**

Motor imagery ability was assessed using mental chronometry during a Purdue Pegboard task. Associations with motor recovery stage, proprioceptive function, and working memory were examined using correlation and multiple regression analyses. An exploratory linear support vector machine analysis was performed to classify participants into groups with higher and lower motor imagery ability using these variables.

**Results:**

Motor imagery ability was significantly associated with motor recovery stage and proprioceptive function in the correlation analyses. However, these associations were no longer independently significant after adjustment in the multiple regression analysis. A linear support vector machine achieved an area under the curve of 0.716, with an accuracy of 0.700, sensitivity of 0.800, and specificity of 0.600. Permutation testing confirmed that the classification performance was above chance.

**Conclusion:**

Motor imagery ability may be related to a combination of motor, sensory, and cognitive functions rather than any single functional domain.

## LAY ABSTRACT

After a stroke, many people experience difficulties in recovering fine motor skills in their hands despite improvements in overall motor function. One contributing factor is difficulty in accurately predicting and imagining movements. In this study, we examined how motor recovery, body sensation, and memory are associated with motor imagery ability. We evaluated 40 individuals with subacute stroke using a simple peg task. Motor recovery and body sensation were associated with motor imagery ability when examined individually. However, no single factor stood out when these factors were considered together. An exploratory analysis further suggested that combining motor recovery, body sensation, and memory allowed classification of motor imagery ability above chance. These findings suggest that motor imagery ability may be related to a combination of motor, sensory, and cognitive functions. Assessing these domains together may help personalize rehabilitation.

Stroke frequently results in upper-extremity motor impairment, which often persists longer than lower-extremity impairment and limits recovery of hand and finger dexterity (1, 2). Within the framework of the International Classification of Functioning, Disability and Health (ICF), impaired hand motor function represents a body function impairment that contributes to activity limitations in tasks requiring fine manual dexterity, such as grasping and object manipulation, thereby restricting independence in daily life. Precise finger movements depend on motor learning processes involving movement prediction and error correction (3–6). Consequently, impaired prediction-error updating may reduce motor learning efficiency and contribute to persistent hand-dexterity deficits (7). Motor imagery (MI) is defined as the internal simulation of movement without actual execution and engages neural processes involved in motor planning (8). During MI, forward internal models are thought to predict the sensory consequences of the imagined movement, thereby allowing motor prediction to occur without overt movement (4, 9). Thus, motor prediction is one of the computational processes underlying MI. Mental chronometry compares imagined and executed movement durations and is widely used as an objective behavioural measure of MI ability (10–13). MI activates motor-related cortical regions, including the supplementary motor area and premotor cortex (14), and may modulate spinal motor neuron excitability (15, 16), suggesting shared neural substrates with motor execution. Because motor recovery and motor learning are active during the subacute stage after stroke, evaluating MI ability may help explain individual differences in motor learning and optimize rehabilitation strategies (17). However, MI ability may involve more than motor recovery and may also be influenced by sensory and cognitive functions.

Motor prediction is related to motor recovery stage, but is not fully explained by it. Studies using F-waves, an index of spinal motor neuron excitability, suggest that waveform diversity decreases after stroke and partially recovers with improvement of finger dexterity (18, 19). However, a previous study found no significant correlation between upper-limb motor function and MI ability (20), suggesting that motor recovery alone cannot fully explain individual differences in MI ability. Proprioceptive function may contribute to MI (21, 22), because internally simulated movements depend on the representation of body position and movement. Furthermore, interventions combining MI with proprioceptive training have been reported to improve motor performance, suggesting that sensory function may influence MI-related rehabilitation outcomes (23). Working memory may support MI by temporarily maintaining and manipulating internally generated motor representations (24–26). Together, these findings suggest that MI ability may be associated with multiple motor, sensory, and cognitive processes rather than with motor recovery alone. However, previous studies have primarily examined these factors individually, and their integrated relationships with MI ability in patients with subacute stroke remain unclear.

Based on this theoretical framework, we hypothesized that MI ability may be associated with motor recovery, proprioceptive function, and working memory rather than any single domain alone. Therefore, the aim of this study was to examine the associations between these factors and MI ability in patients with subacute stroke using correlation and multivariable regression analyses. In addition, an exploratory support vector machine (SVM) analysis was performed to investigate the multidimensional relationships among these variables.

## METHODS

This study was a cross-sectional study involving 4 hospitals. Data collection commenced on 11 March 2024, and was completed on 31 October 2025, based on standardized evaluation procedures at each facility. The inclusion criteria were: (a) first-ever stroke; (b) age ≥ 18 and < 90 years; (c) presence of hemiplegia; and (d) ability to understand the study and provide written informed consent. The exclusion criteria were: (a) left-handed individuals, because of the influence of cerebral hemisphere specialization; (b) individuals with Brunnstrom Recovery Stage (BRS) ≤ 3, for whom voluntary movements were expected to have low reproducibility, making mental chronometry-based MI assessment difficult; (c) individuals with dementia, higher-order cognitive impairments, such as hemispatial neglect, apraxia, and aphasia, or visual field defects based on medical records; (d) individuals with other neurological or orthopaedic conditions; and (e) missing data. An *a priori* power analysis using G*Power 3.1.9.4 (https://www.psychologie.hhu.de/arbeitsgruppen/allgemeine-psychologie-und-arbeitspsychologie/gpower) indicated that 29 participants were required for a two-tailed correlation analysis, assuming α = 0.05, power = 0.80, ρ H0 = 0, and ρ H1 = 0.50. The actual power was 0.813. For the multiple regression analysis, the required sample size was calculated using G*Power 3.1.9.7 with a fixed model with R² deviation from zero. The parameters were set as follows: effect size f² = 0.35, α error probability = 0.05, power = 0.80, and 3 predictors. Based on these settings, the required total sample size was 36 participants, with an actual power of 0.810.

This study was conducted with the approval of the Kansai University of Health Sciences Research Ethics Review Committee (approval number: 24-01). All participants received a thorough explanation of the study’s purpose and methods and provided written informed consent. This study was publicly registered in the UMIN Clinical Trial Registry (registration number: UMIN000053857).

### Outcome measures

**Fig. 1:**
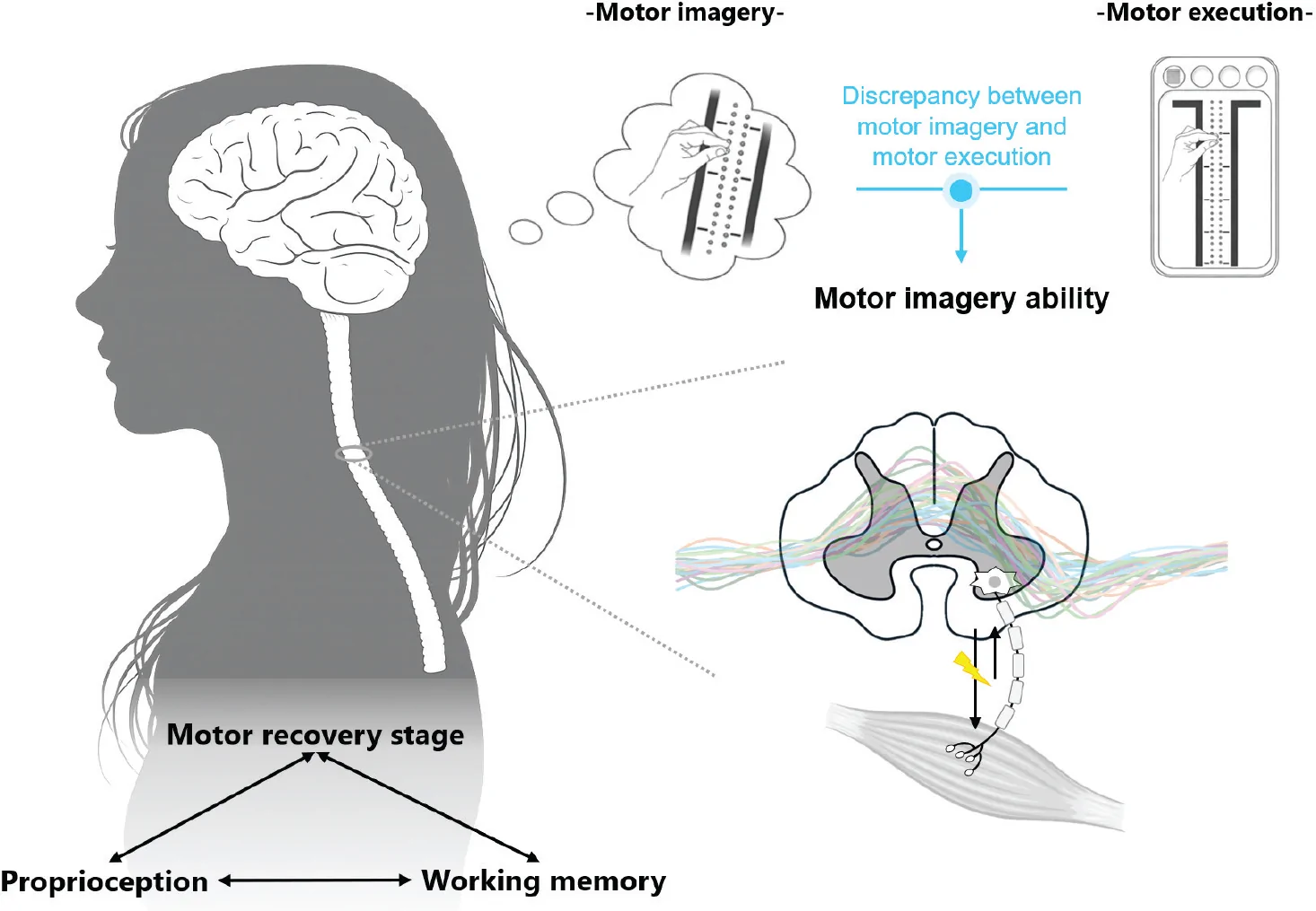
Research design and assessment item configuration. This figure shows the assessment items and analysis configuration used in this study. The assessment items employed were: the Brunnstrom Recovery Stage for the hand as an indicator of motor function recovery stage; the position-sense item of the Stroke Impairment Assessment Set as an indicator of proprioceptive function; the Backward Digit Span as an indicator of working memory; and a mental chronometry-based assessment as an indicator of motor imagery ability. Due to facility and technical limitations, F-wave recording, used as an index of spinal motor neuron excitability, was performed at only 1 facility.

All assessments were conducted within 10 days after enrolment in the study, based on standardized evaluation procedures at each facility. Evaluators completed practice sessions before data collection, followed standardized procedures, and provided consistent instructions to minimize variation in the measurement conditions. The assessment items were: BRS for the hand (BRS-H) to indicate the stage of motor function recovery; the position-sense item of the Stroke Impairment Assessment Set (SIAS-PS) to reflect proprioceptive function; Backward Digit Span (BDS) to reflect working memory; and mental chronometry-based assessment to reflect MI ability. F-waves were recorded at only 1 facility, where the experimental conditions, technical aspects, and facility permission were obtained (Fig. 1).

### Main outcome measures

The BRS was used to assess the motor recovery stage. The BRS classifies motor recovery in the arm, hand, and leg on a 6-point scale (27), with the lowest score being 1 and the highest score being 6; a higher score indicates better motor recovery. In this study, only the hand portion (BRS-H) was used because the aim of this study was to examine the relationship between motor imagery ability and impairment related to voluntary finger movement recovery rather than overall upper-extremity function and activity.

The SIAS was used to assess proprioceptive function. The SIAS classifies 9 types of functional impairments and includes 22 items (28). Because MI involves the internal simulation of movement and associated recall of proprioceptive sensations (29), position sense was measured among the sensory function items. Proprioceptive function was assessed on a 4-point scale, with a minimum score of 0 and a maximum score of 3, with higher score indicating better sensory function.

Working memory was assessed using the BDS, a subtest of the Clinical Assessment for Attention that evaluates phonological loop function. In this task, participants were required to store and manipulate verbal information by recalling digit sequences in reverse order (30), which has been associated with MI ability (25). Digit sequences ranging from 2 to 9 digits were presented auditorily at 1-s intervals. If participants failed both trials at a given sequence length, the test was terminated and the maximum correctly recalled sequence length was recorded as the score.

MI ability was evaluated using a mental chronometry paradigm based on the Purdue Pegboard task. This measure has demonstrated reliability in stroke patients (31). The current assessment procedure and calculation method were based on previously published protocols using the Purdue Pegboard task (12, 32). Participants performed the task with the affected hand, and the number of pins inserted within 60 s was recorded. In the MI condition, participants kinaesthetically imagined the same task for 60 s and reported the number of pins inserted. Participants first performed the imagined peg task using the affected hand once, and then performed the actual peg task using the affected hand once. The absolute difference between the executed and imagined scores was divided by twice the sum of the two scores (32, 33).


$$\frac{\left|Score_{executed}-Score_{imagined}\right|}{2(Score_{executed}+Score_{imagined})}$$

This resulted in values ranging from 0 to 0.5, with values closer to 0 indicating a smaller gap between the executed and imagined scores, signifying higher MI ability. This formula normalizes the absolute discrepancy by the total number of imagined and executed pins, thereby accounting for differences in overall task performance.

### Sub-outcome

F-waves were recorded in a subset of participants to explore spinal motor neuron excitability during MI (see Appendix S1).

### Statistical analysis

Participants with missing data were excluded from the analysis. Normality of the MI ability index was assessed using the Shapiro–Wilk test (*p* = 0.582), and the distribution shape was also assessed visually using histograms and Q–Q plots.

### Primary analyses

The primary analyses consisted of correlation analyses and multiple linear regression analyses to examine the relationships between MI ability and motor recovery stage (BRS-H), proprioceptive function (SIAS-PS), and working memory (BDS). Pearson’s correlation coefficients were calculated to examine the bivariate associations between MI ability and each explanatory variable. Subsequently, multiple linear regression analysis was performed using the enter method, with MI ability as the dependent variable and BRS-H, SIAS-PS, and BDS entered simultaneously as independent variables to evaluate their relative associations while controlling for the influence of the other variables. To examine whether the observed associations were influenced by recovery time, an additional regression model including days since stroke onset as a covariate was also constructed. Multicollinearity was assessed using variance inflation factors (VIFs) and tolerance values. Statistical significance was set at *p* < 0.05, and all these primary analyses were performed using IBM SPSS Statistics version 29.0 (IBM Corp, Armonk, NY, USA).

### Exploratory analyses

For exploratory subgroup analyses, BRS-H, SIAS-PS, and BDS scores were standardized as z-scores to place variables measured on different scales onto a common metric. Participants were then dichotomized into high and low groups using a z-score threshold of 0, corresponding to the sample mean, to facilitate descriptive comparisons between individuals with relatively higher and lower performance within each domain. These subgroup analyses were not used as the primary basis for inference, and no adjustment for multiple testing was applied because they were exploratory in nature. Differences in MI ability between groups were compared using Welch’s *t*-test, which is applicable even when homogeneity of variance cannot be guaranteed. The magnitude of the between-group difference was assessed using Hedges’ *g*.

In a further exploratory analysis, a linear support vector machine (SVM) was used to classify participants into high and low MI ability groups at a 5:5 ratio based on 3 variables: motor recovery stage (BRS-H), proprioceptive function (SIAS-PS), and working memory (BDS). Classification performance was evaluated using 10-fold cross-validation, which provides a good balance between estimation accuracy stability and bias-variance trade-off. This analysis was conducted to examine the multivariate contribution of motor, sensory, and cognitive factors to motor imagery ability. Standardization parameters were estimated from the training data within each fold and then applied to the validation data. Posterior probability estimates were obtained from the linear SVM model implemented in MATLAB. For classification-based performance metrics, including accuracy, sensitivity, and specificity, the participants were assigned to the high or low MI ability group using a predefined probability threshold of 0.5. Performance metrics included accuracy, sensitivity, specificity, and the area under the receiver operating characteristic curve (AUC). The AUC was calculated based on the continuous posterior probability values and was therefore independent of the classification threshold. The multivariate association of each explanatory variable to classification was assessed using the weight coefficient (β) from the linear SVM model. The weight coefficients were evaluated based on the standardized variables. Furthermore, to verify the statistical significance of the classification performance, the same classification analysis procedure was repeated 1,000 times under conditions in which the target variable labels were shuffled randomly, constructing a null distribution of the AUC. *P*-values were calculated based on the position of the observed AUC within the null distribution. These explanatory analyses were performed using SPSS (version 29.0.0.0; IBM, Armonk, NY, USA) and MATLAB R2024a (MathWorks, Natick, MA, USA), with a significance level set at 5%.

### Sub-analysis

Exploratory F-wave analysis was performed in a subset of participants (see Appendix S1).

## RESULTS

### Subject characteristics

**Fig. 2:**
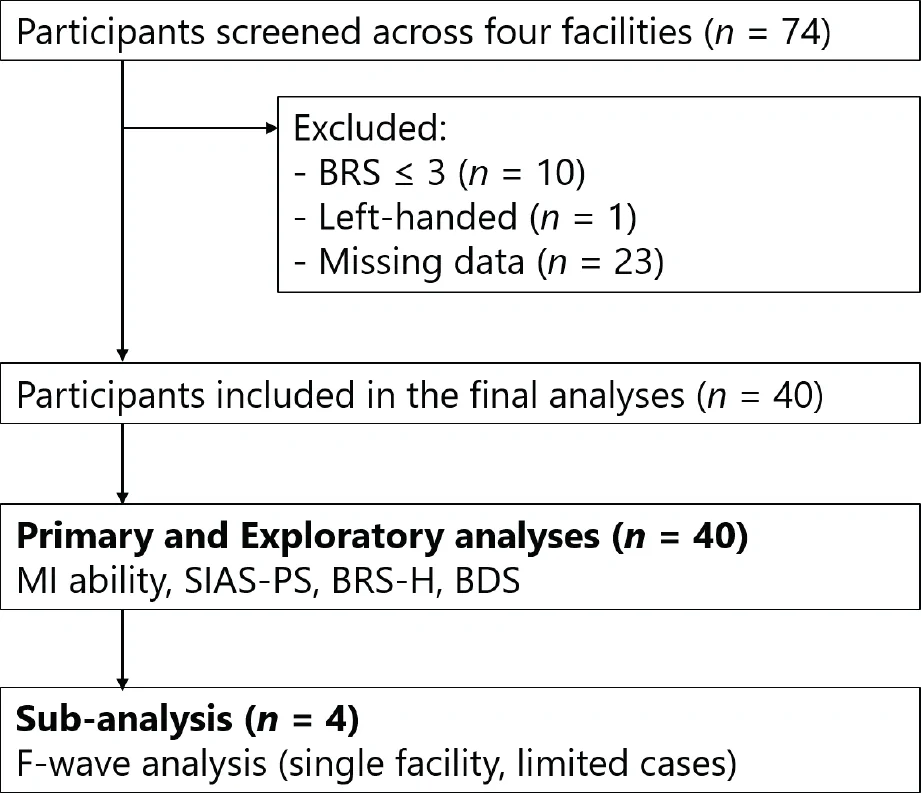
Study participant selection flow and analysis structure. Seventy-four participants with subacute stroke were enrolled across 4 facilities. After excluding cases with Brunnstrom Recovery Stage (BRS) ≤3, left-handedness, or missing data, 40 participants were ultimately included in the analysis. The primary analyses examined motor imagery ability as the dependent variable, with BRS for the hand (BRS-H), the position-sense item of the Stroke Impairment Assessment Set (SIAS-PS), and Backward Digit Span (BDS) as explanatory variables. Additionally, F-wave analysis was performed as an explanatory analysis in a subset of participants at a single institution. MI: motor imagery.

Seventy-four subacute stroke patients were enrolled in this study (mean [SD] age, 68.4 [13.2] years; 41 males; mean [SD] time since onset, 81.1 [47.8] days; haemorrhagic stroke, 33 cases; ischaemic stroke, 41 cases; right hemiplegia, 36 cases; left hemiplegia, 38 cases). Participants excluded from analysis were those with BRS ≤3 (*n* = 10), left-handed individuals (*n* = 1), and those with missing data (*n* = 23), resulting in a final analysis group of 40 patients (Fig. 2). The characteristics of the analysis group are presented in Table I.

**Table I:** Participant characteristics and exploratory subgroup classification

Characteristic/Measure	*n* = 40			
Age, year	71.0 (12.9)			
Sex				
Male	20 (50%)			
Female	20 (50%)			
Type of stroke				
Infarction	23 (57.5%)			
Haemorrhage	17 (42.5%)			
Paretic side				
Right	19 (47.5%)			
Left	21 (52.5%)			
Time since stroke, days	76.3 (49.7)			
Exploratory subgroup classification				
	Original score, mean (SD)	Subgroup mean (SD)	Low group (*n*)	High group (*n*)
BRS-H	5.4 (0.7)	Low, 4.7 (0.4)/High, 6.0(0.0)	21	19
SIAS-PS	2.6 (0.7)	Low, 1.5 (0.7)/High, 3.0(0.0)	11	29
BDS	3.3 (1.9)	Low, 0.5 (1.0)/High, 4.3(0.6)	11	29

Data are presented as mean (SD) or *n* (%), unless otherwise indicated. BRS-H: Brunnstrom Recovery Stage for the hand; SIAS-PS: position-sense item of the Stroke Impairment Assessment Set; BDS: Backward Digit Span.

### Primary results

*Correlation analyses.* Pearson’s correlation analyses showed that MI ability was significantly associated with motor recovery stage (BRS-H; *r* = −0.316, *p* = 0.047) and proprioceptive function (SIAS-PS; *r* = −0.422, *p* = 0.007), whereas no significant correlations were observed with working memory (BDS; *r* = −0.174, *p* = 0.282) or days since stroke onset (*r* = 0.003, *p* = 0.983) (Table II).

**Table II:** Primary analyses examining factors associated with motor imagery ability

Variable	Pearson’s *r* (vs MI ability).	*p*-value	Model 1 β	95% CI (B)	*p*-value	VIF	Model 2 β	95% CI (B)	*p*-value	VIF
BRS-H	–0.316	0.047	–0.147	–0.086 to 0.035	0.402	1.384	–0.150	–0.088 to 0.036	0.401	1.395
SIAS-PS	–0.422	0.007	–0.337	–0.112 to 0.002	0.060	1.385	–0.334	–0.112 to 0.004	0.067	1.394
BDS	–0.174	0.282	–0.163	–0.030 to 0.009	0.278	1.009	–0.168	–0.031 to 0.009	0.278	1.042
Days poststroke	0.003	0.983					0.028	–0.001 to 0.001	0.853	1.039
Model summary			R² = 0.217 (Adj R² = 0.152), F = 3.326, *p* = 0.030				R² = 0.218 (Adj R² = 0.128), F = 2.437, *p* = 0.065			

BRS-H: Brunnstrom Recovery Stage for the hand; SIAS-PS: position-sense item of the Stroke Impairment Assessment Set; BDS: Backward Digit Span; CI: confidence interval; VIF: variance inflation factor. Lower motor imagery ability index values indicate better motor imagery ability.

Model 1: Multiple linear regression model including BRS-H, SIAS-PS, and BDS as independent variables. Model 2: Multiple linear regression model including BRS-H, SIAS-PS, BDS, and days since stroke onset as independent variables.

*Multiple regression analyses.* In Model 1, multiple linear regression analysis demonstrated that the overall model was significant (R² = 0.217, adjusted R² = 0.152, *F*(3,36) = 3.326, *p* = 0.030). However, none of the independent variables reached significance after adjustment for the other variables (BRS-H: β = −0.147, *p* = 0.402; SIAS-PS: β = −0.337, *p* = 0.060; BDS: β = −0.163, *p* = 0.278). No evidence of problematic multicollinearity was observed (all variance inflation factors <1.4) (see Table II). In Model 2, which additionally included days since stroke onset, the overall model was no longer significant (R² = 0.218, adjusted R² = 0.128, *F*(4,35) = 2.437, *p* = 0.065). Days since stroke onset was not independently associated with MI ability (β = 0.028, *p* = 0.853), and inclusion of this variable produced only a minimal change in the model’s explanatory power (Table II).

### Exploratory results

**Fig. 3:**
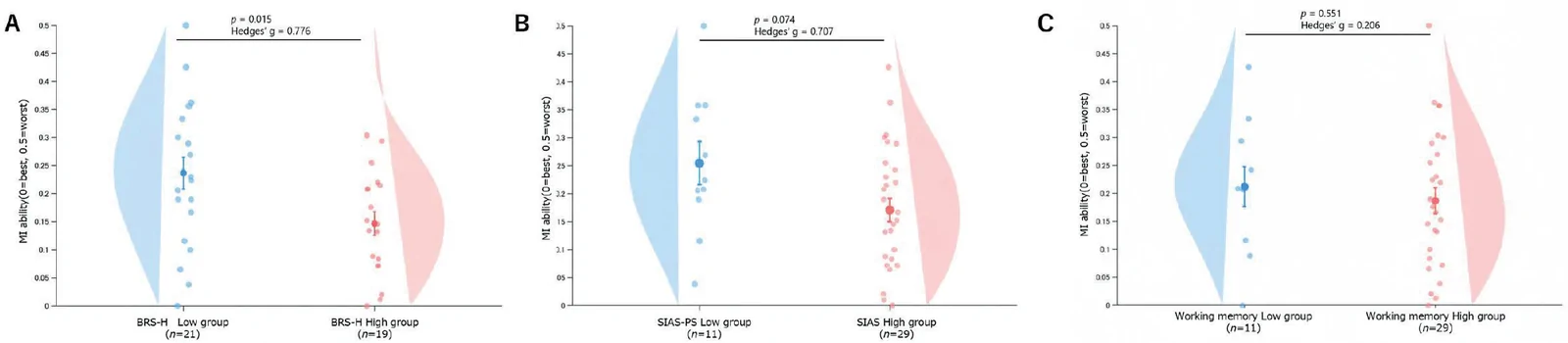
Intergroup comparison of motor imagery (MI) ability by motor recovery stage, sensory function, and working memory. (A). Comparison of MI ability based on Brunnstrom Recovery Stage for the hand (BRS-H). Each point represents individual data, with the central plot indicating the mean and standard error. The background distribution represents the data distribution for each group. The MI ability index (0 = best, 0.5 = worst) was significantly higher in the low BRS-H group than in the high BRS-H group, indicating poorer MI ability in the low BRS-H group (B). Comparison of the MI ability index based on the position-sense item of the Stroke Impairment Assessment Set (SIAS-PS); no significant between-group differences were observed (C). Comparison of the MI ability index based on the Backward Digit Span; no significant between-group differences were observed.

*MI ability by each explanatory variable.* A significant difference was observed between the high (*n* = 19) and low (*n* = 21) groups for the BRS-H (*t*[35.99] = 2.545, *p* = 0.015). The mean difference was 0.0896 (95% confidence interval [0.018, 0.161]). The effect size was moderate to large (Hedges’ *g* = 0.776; Fig. 3A). The between-group difference for the SIAS-PS between the high (*n* = 29) and low (*n* = 11) groups did not reach significance (*t*[16.11] = 1.913, *p =* 0.074). The mean difference was 0.0837 (95% confidence interval [−0.009, 0.176]), with a moderate effect size (Hedges’ *g* = 0.707; Fig. 3B). The difference between the high (*n* = 29) and low (*n* = 11) BDS groups also did not reach significance (*t*[18.90] = 0.607, *p* = 0.551). The mean difference was 0.0256 (95% confidence interval [–0.063, 0.114]). The effect size was small (Hedges’ *g* = 0.206; Fig. 3C).

**Fig. 4:**
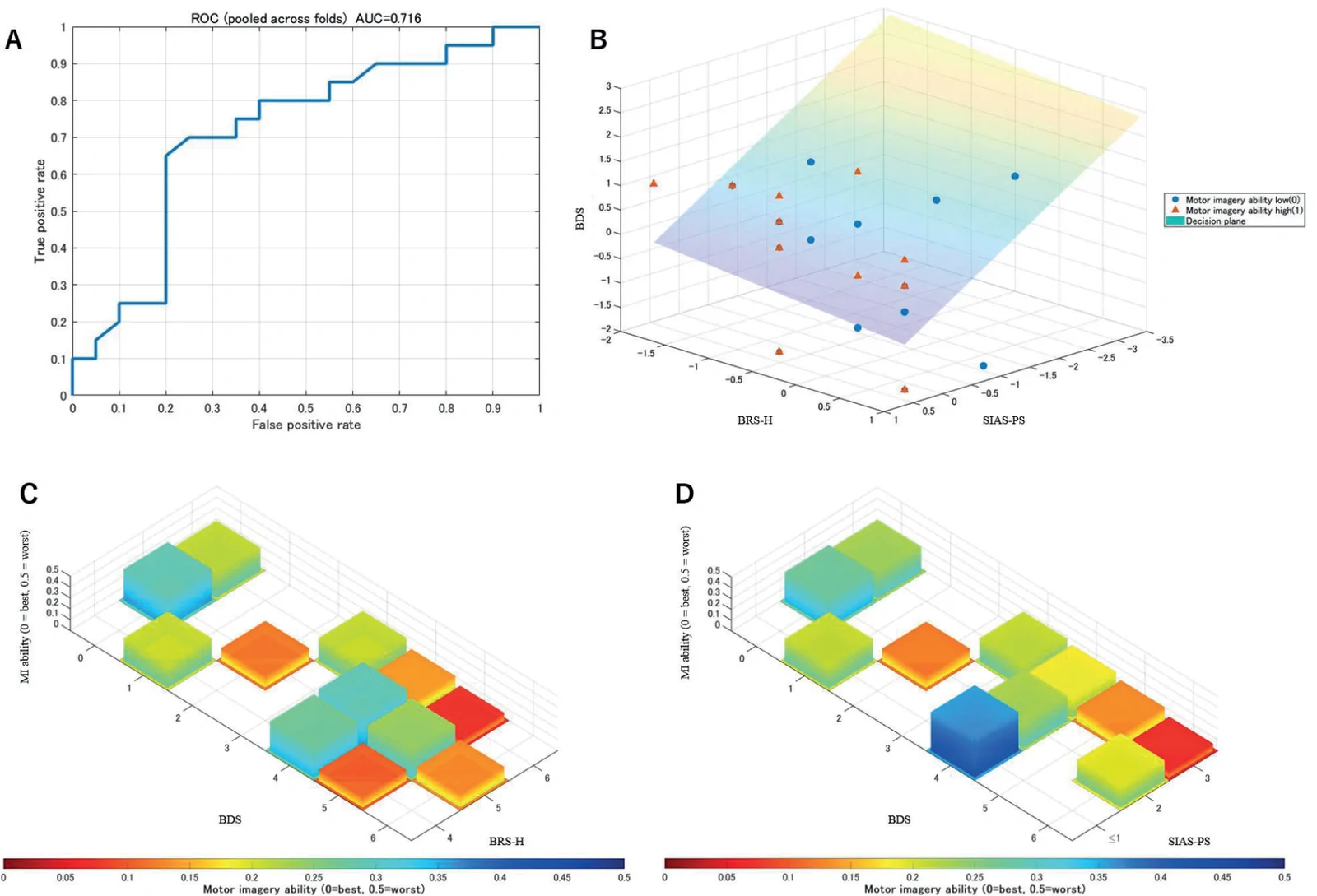
Receiver operating characteristic (ROC) curve and decision plane for support vector machine (SVM) classification of motor imagery (MI) ability, with descriptive heatmaps. (A). ROC curve for classifying the high and low MI ability groups using a linear SVM. The curve was generated by aggregating prediction results across all folds from 10-fold cross-validation, yielding an area under the curve (AUC) of 0.716 (B). Three-dimensional representation of the linear support vector machine (SVM) model using Brunnstrom Recovery Stage for the hand (BRS-H), the position-sense item of the Stroke Impairment Assessment Set (SIAS-PS), and Backward Digit Span (BDS). The axes show the standardized values of the 3 variables. Each point represents an individual participant, with red triangles indicating the high MI ability group and blue circles indicating the low MI ability group. The semi-transparent plane represents the decision boundary of the fitted linear SVM model and is visually inclined primarily along the BDS axis, followed by the SIAS-PS axis (C). Three-dimensional heatmap showing the median MI ability index according to combinations of Brunnstrom Recovery Stage for the hand (BRS-H) and Backward Digit Span (BDS). The vertical axis represents the MI ability index (0 = optimal, 0.5 = suboptimal). Each tile represents the median MI ability of the corresponding subgroup. Red indicates better MI ability and blue indicates poorer MI ability (D). Three-dimensional heatmap showing the median MI ability index according to combinations of the position-sense item of the Stroke Impairment Assessment Set (SIAS-PS) and Backward Digit Span (BDS). The vertical axis represents the MI ability index (0 = optimal, 0.5 = suboptimal). Each tile represents the median MI ability of the corresponding subgroup. Red indicates better MI ability and blue indicates poorer MI ability.

*Classification using linear SVM.* The results of 10-fold cross-validation showed a mean (SD) accuracy rate of 0.700 (0.230), sensitivity of 0.800 (0.350), specificity of 0.600 (0.394), and an AUC of 0.716 (0.297) (Fig. 4A). The confusion matrix showed that 16 out of 20 cases in the high MI ability group and 12 out of 20 cases in the low MI ability group were correctly classified. Furthermore, the significance of the AUC was confirmed by a 1,000-replication permutation test (*p* = 0.032), indicating that this classification performance was not due to chance. The linear SVM weight coefficients were largest for the BDS (0.831), followed by SIAS-PS (0.662) and BRS-H (0.156) (Fig. 4B). To facilitate interpretation of the classification model, the absolute values of the linear SVM weight coefficients were normalized by dividing each coefficient by the sum of all coefficients. The resulting values were expressed as percentages representing the normalized feature weights.

To descriptively visualize MI ability across combinations of motor recovery stage (BRS-H), proprioceptive function (SIAS-PS), and working memory (BDS), a three-dimensional heatmap was created that showed representative MI ability values for each combination of the indicators. In the BRS-H–BDS heatmap, higher BDS scores tended to correspond to better MI ability at the moderate stage of motor recovery (Fig. 4C). Furthermore, the heatmap visually showed different patterns of MI ability across combinations of SIAS-PS and BDS (Fig. 4D).

*F-wave indicators.* In a subset of participants (*n* = 4), F-wave indices during MI showed heterogeneous patterns across cases (see Appendix S1).

## DISCUSSION

We conducted a multicentre cross-sectional study to examine the relationships between motor recovery stage, proprioceptive function, working memory, and MI ability in individuals with subacute stroke. Pearson’s correlation analyses demonstrated significant associations of MI ability with motor recovery stage and proprioceptive function. In contrast, although the overall multiple regression model was significant, none of the individual explanatory variables remained independently associated with MI ability after adjustment for the other variables. Exploratory analyses further showed that classification based on the combination of motor recovery stage, proprioceptive function, and working memory distinguished high and low MI ability above chance level. These findings suggest that MI ability is associated with multiple functional domains and provide a basis for further examining whether and how these domains may interact to support motor imagery after stroke.

### Integrated associations among motor, sensory, and cognitive factors

The present findings suggest that motor imagery (MI) ability in individuals with subacute stroke may not be sufficiently explained by any single motor, sensory, or cognitive domain. Although motor recovery stage and proprioceptive function were significantly associated with MI ability in the correlation analyses, these associations were no longer independently significant after adjustment for the other variables in the multiple regression model. This pattern suggests that the observed relationships may partly reflect interrelationships among motor recovery, proprioceptive function, and working memory rather than independent effects of each domain.

This interpretation is consistent with contemporary theories of motor control, which propose that motor imagery is supported by internal forward models that integrate motor commands with predicted sensory consequences to simulate movement without overt execution (9). From this perspective, accurate motor imagery depends on the coordinated integration of motor, sensory, and cognitive information rather than on any single functional system. Neuroimaging studies have also demonstrated that motor imagery recruits distributed cortical networks involved in motor planning, sensorimotor processing, and cognitive control (14), supporting the concept that MI is inherently a multidimensional process. Therefore, the present findings are consistent with the possibility that MI ability emerges from the interaction of multiple functional domains rather than the independent influence of any single factor.

### Potential roles of motor recovery, proprioception, and working memory

The present findings may provide further insight into the complementary roles of motor recovery, proprioception, and working memory in motor imagery after stroke. Motor recovery may provide the motor repertoire available for internally simulating voluntary movements. However, previous studies have shown that motor recovery alone does not fully explain motor imagery ability, with substantial inter-individual variability observed even among patients with similar levels of motor impairment (20, 34). Consistent with these findings, motor recovery stage was associated with MI ability in the correlation analysis but was not independently associated with MI ability after adjustment for proprioceptive function and working memory.

Proprioceptive function may contribute to motor imagery by improving the accuracy of internally predicted sensory consequences through sensorimotor integration (21). Previous studies have reported that sensory impairment after stroke is associated with reduced motor imagery accuracy and impaired mental chronometry (22, 35), whereas interventions incorporating proprioceptive input may facilitate motor imagery performance (23). Although proprioceptive function was not independently associated with MI ability in the multiple regression analysis, it consistently demonstrated the largest absolute standardized regression coefficient, the strongest absolute correlation coefficient, and a moderate effect size in the exploratory subgroup analysis. Taken together, these findings suggest that proprioceptive function may represent an important component of motor imagery, although its independent contribution could not be confirmed in the present study.

Working memory may complement these motor and sensory processes by maintaining and manipulating internally generated motor representations during mental simulation (24–26, 36). Neuroimaging studies have demonstrated that motor imagery recruits distributed cortical networks involving motor planning and cognitive control regions (14), supporting the concept that successful motor imagery depends on coordinated motor, sensory, and cognitive processing rather than a single functional domain. Consistent with this interpretation, none of the explanatory variables was independently associated with MI ability in the multiple regression analysis, whereas the exploratory SVM analysis demonstrated that combining motor recovery stage, proprioceptive function, and working memory enabled classification of MI ability above chance level. Collectively, these findings suggest that considering motor recovery stage, proprioceptive function, and working memory together may provide a useful framework for understanding variability in MI ability after stroke. However, the specific mechanisms by which these domains interact remain to be established in future longitudinal and experimental studies.

### Excitability of spinal motor neurons during MI: insights from F-wave analysis

F-wave measurements were included as an exploratory physiological readout to examine how multivariate motor recovery, proprioception, and working memory profiles might be reflected at the spinal level during MI. Although heterogeneous patterns of F-wave responses were observed across cases, the small sample size and differences in measurement timing limit a definitive interpretation. These findings should therefore be considered preliminary (see Appendix S1).

### Study limitations

Because this was a multicentre cross-sectional study, it cannot establish causal relationships among sensory function, working memory, motor recovery, and MI ability. The findings reflect associations only and require validation in longitudinal or interventional studies. Individuals with severe motor impairment (BRS-H ≤3) were excluded from this study. Therefore, the present findings may not be generalizable to patients with more severe upper-extremity motor impairment, and future studies should examine whether similar relationships are observed across a broader range of motor recovery stages. Also, potential selection bias due to the exclusion of participants with missing data cannot be ruled out. In addition, the exploratory subgroup analyses based on z-score dichotomization resulted in relatively small and imbalanced subgroup sizes for some variables. Therefore, the estimated effect sizes and between-group comparisons should be interpreted cautiously because they may have been influenced by limited statistical stability. Furthermore, the support vector machine analysis was exploratory. Although internal validation was performed, the relatively large variability in classification performance indicates limited model stability. External validation using larger independent cohorts is required before these findings can be generalized. MI ability assessed by mental chronometry may have been influenced by attention and motor performance, limiting strict differentiation between MI and execution deficits. Sensory function and working memory were assessed using limited clinical measures that may not have fully captured the multidimensional nature of these functions. In addition, motor recovery was assessed only using the Brunnstrom Recovery Stage for the hand (BRS-H). Comprehensive upper-extremity assessments, such as the Fugl-Meyer Assessment and the Action Research Arm Test, were not included. Therefore, the present findings should be interpreted as reflecting impairment related to voluntary finger movement recovery rather than overall upper-extremity motor function. The F-wave analysis was exploratory and based on a small sample; therefore, the F-wave results should be interpreted as preliminary and hypothesis-generating. Furthermore, this study focused on motor recovery stage, proprioceptive function, and working memory based on the proposed theoretical framework. However, other factors that may influence motor imagery ability after stroke, including lesion location, attentional function, executive function, body representation, and the severity of sensory impairment, were not evaluated. These factors may also contribute to the variability in motor imagery ability observed after stroke and should be incorporated into future studies to achieve a more comprehensive understanding of the mechanisms underlying motor imagery.

In conclusion, we examined the relationships among motor recovery stage, proprioceptive function, working memory, and MI ability in individuals with subacute stroke. The findings suggest that MI ability is associated with multiple motor, sensory, and cognitive factors rather than a single functional domain. These results support further investigation of rehabilitation strategies that consider proprioceptive function and working memory in addition to motor recovery. For example, training that involves holding and updating proprioceptive information (37, 38) may be a target for future intervention studies.

## Supplementary Material


