# Supplementary material for: CLINICAL FACTORS ASSOCIATED WITH MOTOR IMAGERY ABILITY IN INDIVIDUALS WITH SUBACUTE STROKE
Source: J Rehabil Med. 2026 Aug 17;58:46042. doi: 10.2340/jrm.v58.46042 (PMC13491540; doi:10.2340/jrm.v58.46042)
Supplement: Supplementary file 1 [file JRM-58-46042-s1.pdf]

Methods

F-waves were used to evaluate changes in the excitability of spinal motor neurons. An F-wave is a compound muscle action potential recorded in the corresponding muscle that is generated when a retrograde impulse, resulting from maximal electrical stimulation applied to mixed nerve fibers in the periphery, travels up an axon and is reactivated in a spinal anterior horn cell, then travels down the axon as an anterograde impulse. F-waves were recorded using the Neuropack S3 evoked electromyography system (Nihon Kohden Corporation, Tokyo, Japan). The recording conditions were as follows: the stimulating electrode was placed over the median nerve at the paralyzed wrist; the recording electrode was placed over the thenar muscle group; the reference electrode was placed dorsally over the head of the left first metacarpal bone; and the ground electrode was placed centrally on the palmar surface of the left forearm. A bipolar bar electrode (Au) was used for the stimulation electrode, a 10-mm diameter EEG cup electrode (Ag/AgCl) for the reference/recording electrode, and a 30-mm diameter plate ground electrode (stainless) for the ground electrode. Stimulation conditions consisted of 30 stimuli at 0.5 Hz with a maximum upper stimulation intensity and a duration of 0.2 ms. F-waves were measured during rest and MI. Changes in the F/M amplitude ratio and occurrence frequency during MI relative to rest were calculated and used as indices of spinal motor neuron excitability.(1) Furthermore, to confirm the presence of repetitive F-waves, which are thought to be related to finger dexterity in stroke patients, proprietary software was used to detect F-waves and verify the similarity of their waveforms.

Sub-analysis

For a subset of cases in which F-wave data were obtainable, the relationship between F-wave indices and MI ability was explored, providing a mechanistic consideration of the relationship between spinal motor neuron excitability and MI capability. However, due to equipment and technical limitations, the number of cases analyzed for F-waves was limited ($n = 4$). Therefore, this analysis was not used for the main conclusions of this study and is positioned as supplementary results.

Results

The four subacute stroke hemiplegia patients (Table SI) who underwent F-wave measurement had the following characteristics: Patients A, B, and C had a BRS-H score of 4, indicating they were at a similar stage of motor recovery; however, Patient D had a BRS-H score of 6, indicating a more advanced stage of motor recovery. The SIAS-PS scores were 3 points for Patients A and D, 2 points for Patient B, and 1 point for Patient C, with Patient C showing the most severe impairment. The BDS score was 5 for Patient A and 4 for Patients B, C, and D. The MI ability scores were 0.10 for Patient A, 0.04 for Patient B, 0.50 for Patient C, and 0.13 for Patient D.

In Patient A, the frequency of F-wave appearance increased from 77% at rest to 97% during MI. Conversely, the amplitude F/M ratio decreased from 3.06% at rest to 2.03% during MI. In Patient B, the F-wave occurrence rate showed no change, remaining at 93% at rest and during MI. The amplitude F/M ratio decreased from 3.55% at rest to 2.78% during MI. In contrast to Patients A and B, in Patient C, the F-wave occurrence frequency increased from 90% at rest to 100% during MI, and the amplitude F/M ratio also rose from 1.88% at rest to 2.33% during MI. In Patient D, the F-wave occurrence frequency during MI showed only a slight increase, from 93% at rest to 96% during MI; the amplitude F/M ratio decreased slightly from 3.29% at rest to 2.98% during MI, showing no significant variation compared to rest. Repeater F-waves represent a phenomenon where identical or highly similar F-wave patterns appear repeatedly, suggesting that the firing patterns of spinal anterior horn cells are fixed and selective. Waveform analysis revealed no occurrence of repeater F-waves in any patient either at rest or during MI (Fig. S1).

Discussion

On the basis of primary analysis demonstrating the differential contributions of motor recovery stage, proprioceptive function, and cognitive resources to MI ability, F-wave measurements were used as a secondary physiological readout to examine how these profiles might be reflected at the spinal level during MI. In Patient D (BRS-H = 6), both the occurrence frequency of F-waves during MI and the change in the F/M amplitude ratio were small, showing little difference from rest. In contrast, Patients A–C (BRS-H = 4) showed variable F-wave responses during MI. Previous studies have reported that spinal motor neuron excitability can change during MI even in stroke patients.(2) However, motor recovery stage alone may not determine spinal responses during MI, as motor function and MI task performance do not necessarily correlate.(3) Furthermore, since F-wave indices reportedly show a minimal change during highly accurate MI,(4) the small changes observed in Patient D (BRS-H = 6) may be consistent with relatively stable MI performance.

Focusing next on proprioceptive function, only Patient C showed a marked decline (SIAS-PS = 1), and this case also exhibited the lowest MI ability. In contrast, proprioceptive function was preserved in Patients A and D (SIAS-PS = 3), while Patient B showed a moderate decline (SIAS-PS = 2). MI is an internal simulation process involving the recall of motor commands and the prediction of sensory outcomes without requiring new sensory input.(5) Mental chronometry is reportedly impaired in stroke patients with sensory deficits,(6) suggesting that degraded sensory representation may interfere with the establishment of MI. From this perspective, the reduced proprioceptive function in Patient C may partly explain the lower MI ability observed. In this patient, the F-wave responses during MI were characterized by increases in the occurrence frequency and amplitude F/M ratio. Although the mechanism cannot be determined from the present observations, this pattern may reflect the altered modulation of spinal motor neuron excitability during MI in the presence of impaired sensory representation. Previous reports have shown that in conditions such as spasticity, F-wave occurrence frequency can increase without a corresponding increase in maximal

amplitude.(1) Therefore, the concurrent increase observed in both indices in this patient suggests that the response pattern during MI may differ from the typical excitability changes reported in spastic conditions. Since the peg task requires a relatively complex sensorimotor representation, low MI ability may lead to less selective descending inputs and more variable spinal responses. Consistent with this interpretation, no repeater F-waves were observed during rest or MI, and the large-amplitude waveforms seen during rest were not consistently present during MI. Although descriptive, these findings may indicate that when MI is insufficiently established, spinal responses during MI become more heterogeneous.

Finally, focusing on working memory, only Patient A showed a relatively high score (BDS = 5), whereas the other patients had comparable scores (BDS = 4). No clear relationship was observed between working memory capacity and MI ability. However, Patient A showed a relatively larger increase in F-wave occurrence frequency during MI. Working memory reportedly influences responsiveness to MI interventions,(7) but is not the sole determinant of performance accuracy.(8) Higher working memory capacity may facilitate the maintenance of motor representations and sustained attention during MI,(9) potentially contributing to the initiation of central drive. However, the present observations suggest that working memory alone does not determine MI ability and may instead play a supportive role in MI processing.

References

1. Eisen A, Odusote K. Amplitude of the F wave: a potential means of documenting spasticity. Neurology 1979; 29: 1306–1309. DOI: 10.1212/wnl.29.9_part_1.1306

2. Naseri M, Petramfar P, Ashraf A. Effect of motor imagery on the F-wave parameters in hemiparetic stroke survivors. Ann Rehabil Med 2015; 39: 401–408. DOI: 10.5535/arm.2015.39.3.401

3. Kemlin C, Moulton E, Samson Y, Rosso C. Do motor imagery performances depend on the side of the lesion at the acute stage of stroke? Front Hum Neurosci 2016; 10: 321. DOI: 10.3389/fnhum.2016.00321

4. Fukumoto Y, Bizen H, Todo M, Yoshida N, Suzuki T. The combined effects of motor imagery and motor practice are influenced by differences in working memory function: Examination of brain, spinal cord, and muscle performance data. IBRO Neurosci Rep 2025; 19: 210–222. DOI: 10.1016/j.ibneur.2025.06.016

5. Jeannerod M. Neural simulation of action: a unifying mechanism for motor cognition. Neuroimage 2001; 14: S103–9. DOI: 10.1006/nimg.2001.0832

6. Liepert J, Greiner J, Nedelko V, Dettmers C. Reduced upper limb sensation impairs mental chronometry for motor imagery after stroke: clinical and electrophysiological findings: Clinical and electrophysiological findings. Neurorehabil Neural Repair 2012; 26: 470–478. DOI: 10.1177/1545968311425924

7. Malouin F, Belleville S, Richards CL, Desrosiers J, Doyon J. Working memory and mental practice outcomes after stroke. Arch Phys Med Rehabil 2004; 85: 177–183. DOI: 10.1016/s0003-9993(03)00771-8

8. Anguera JA, Bernard JA, Jaeggi SM, Buschkuehl M, Benson BL, Jennett S, et al. The effects of working memory resource depletion and training on sensorimotor adaptation. Behav Brain Res 2012; 228(1): 107–115. DOI: 10.1016/j.bbr.2011.11.040

9. Curtis CE, D'Esposito M. Persistent activity in the prefrontal cortex during working memory. Trends Cogn Sci 2003; 7(9): 415–423. DOI: 10.1016/s1364-6613(03)00197-9

Fig. S1. F-wave analysis

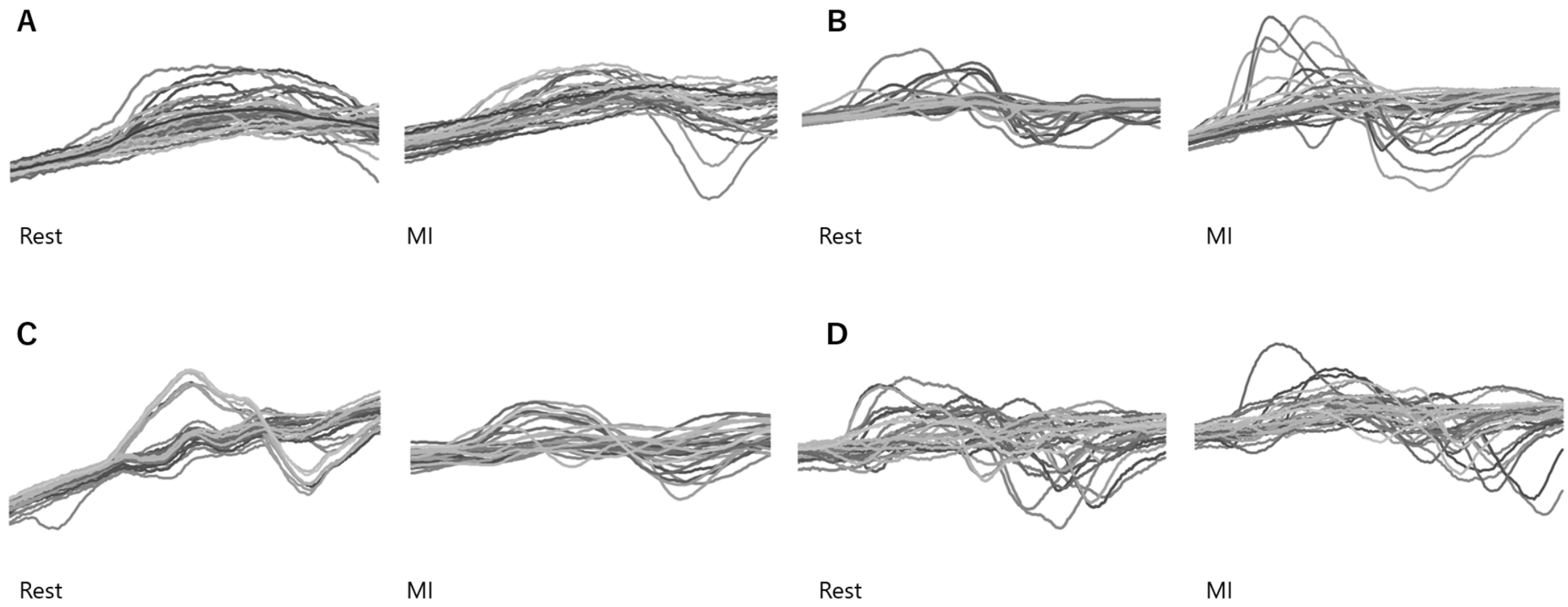

F-wave indices and waveform superimposition during rest and motor imagery (MI) for four patients (A–D) with differing recovery stages, proprioceptive function, and working memory. The grayscale traces represent individual recorded waveforms. Repeater F-waves were not observed in any patient during the 60 stimuli recorded across rest and MI . (A) Brunnstrom Recovery Stage for the hand (BRS-H) = 4, position-sense item of the Stroke Impairment Assessment Set (SIAS-PS) = 3, Backward Digit Span (BDS) = 5, with relatively good MI ability (0.10). F-wave occurrence frequency increased from 77% at rest to 97% during MI, while the F/M amplitude ratio decreased from 3.06% at rest to 2.03% during MI. (B) BRS-H = 4, SIAS-PS = 2, BDS = 4; MI ability was good (0.04). F-wave occurrence rate was unchanged between rest and MI (93% in both conditions); F/M amplitude ratio decreased from 3.55% at rest to 2.78% during MI. (C) BRS-H = 4, but SIAS-PS = 1 with markedly impaired proprioceptive function, with an MI ability index of 0.50, indicating the poorest. F-wave occurrence frequency increased from 90% at rest to 100% during MI, and the F/M amplitude ratio rose from 1.88% at rest to 2.33% during MI. (D) BRS-H = 6, SIAS-PS = 3, BDS = 4, with relatively preserved MI ability (0.13). The F-wave occurrence frequency increased slightly from 93% at rest to 96% during MI, while the F/M amplitude ratio decreased marginally from 3.29% at rest to 2.98% during MI.

Table SI. Basic information for patients included in the F-wave analysis

Participant	BRS-H	SIAS-PS	BDS	MI ability
A	4	3	5	0,1
B	4	2	4	0,04
C	4	1	4	0,5
D	6	3	4	0,13

BRS-H, Brunnstrom Recovery Stage for the hand; MI, motor imagery; SIAS-PS, Proprioception Sensory function of the Stroke Impairment Assessment Set.
